# Radiotheranostics - Precision Medicine in Nuclear Medicine and Molecular Imaging

**DOI:** 10.7150/ntno.64141

**Published:** 2022-01-01

**Authors:** Heying Duan, Andrei Iagaru, Carina Mari Aparici

**Affiliations:** Department of Radiology, Division of Nuclear Medicine and Molecular Imaging, Stanford University, Stanford, CA, USA

## Abstract

'See what you treat and treat what you see, at a molecular level', could be the motto of theranostics. The concept implies diagnosis (imaging) and treatment of cells (usually cancer) using the same molecule, thus guaranteeing a targeted cytotoxic approach of the imaged tumor cells while sparing healthy tissues. As the brilliant late Sam Gambhir would say, the imaging agent acts like a 'molecular spy' and reveals where the tumoral cells are located and the extent of disease burden (diagnosis). For treatment, the same 'molecular spy' docks to the same tumor cells, this time delivering cytotoxic doses of radiation (treatment). This duality represents the concept of a 'theranostic pair', which follows the scope and fundamental principles of targeted precision and personalized medicine.

Although the term *theranostic* was noted in medical literature in the early 2000s, the principle is not at all new to nuclear medicine. The first example of theranostic dates back to 1941 when Dr. Saul Hertz first applied radioiodine for radionuclide treatment of thyroid cells in patients with hyperthyroidism. Ever since, theranostics has been an integral element of nuclear medicine and molecular imaging. The more we understand tumor biology and molecular pathology of carcinogenesis, including specific mutations and receptor expression profiles, the more specific these 'molecular spies' can be developed for diagnostic molecular imaging and subsequent radionuclide targeted therapy (radiotheranostics). The appropriate selection of the diagnostic and therapeutic radionuclide for the 'theranostic pair' is critical and takes into account not only the type of cytotoxic radiation emission, but also the linear energy transfer (LET), and the physical half-lives. Advances in radiochemistry and radiopharmacy with new radiolabeling techniques and chelators are revolutionizing the field. The landscape of cytotoxic systemic radionuclide treatments has dramatically expanded through the past decades thanks to all these advancements. This article discusses present and promising future theranostic applications for various types of diseases such as thyroid disorders, neuroendocrine tumors (NET), pediatric malignancies, and prostate cancer (PC), and provides an outlook for future perspectives.

## Introduction

The term *theranostic* became increasingly popular since the early 2000s and its publications have been rapidly increasing ever since. Theranostic is a portmanteau of the Greek words *therapo* and *gnosis*, translating to *therapy* and *knowing*, i.e., diagnostics 1, 2. There is a constant debate whether 'theranostics' or 'theragnostics' should be used, however both spellings are acknowledged. The principle of theranostic is to identify the right molecular probe (diagnostic/therapeutic) for the right patient in order to maximize subsequent treatment outcome while minimizing toxicity. It stratifies upfront future responders from non-responders, hence preventing unnecessary treatments, sparing patients from the usual trial and error approach, and saving unnecessary drug costs for the healthcare system. The theranostic concept applies to many different areas of science depending on the platform used to convey the main principle. In that sense, theranostics could be further categorized as: radiotheranostic, nanotheranostic, magnetotheranostic and optotheranostic, by using radionuclides, nanoparticles, magnetic particles and optical probes, respectively 3, 4. Out of all the possible categories, radiotheranostic has been so far the only one widely adopted and integrated clinically, and therefore commonly referred to as theranostics. Radiotheranostics uses low penetration radiation emitted from radionuclides to deposit high levels of energy in the nucleus of the targeted cells to induce DNA strand breaks and activate programmed cell death. The concept of radiotheranostics has been clinically adopted for over 80 years now. Among the earliest examples of theranostic is the use of Phosphorus-32 (^32^P) for leukemia in 1941 5, and Iodine-131 (^131^I) for Graves' disease published by Saul Hertz in 1942 6, and subsequently thyroid cancer in 1946 7. Radioiodine therapies became one of the cornerstone treatments in nuclear medicine and is markedly used every day worldwide. Radiotheranostic complies with the ultimate concept of personalized medicine by using paired diagnostic/therapeutic radionuclide probes for the selective and targeted diagnosis and treatment of specific (usually cancer) cells, tailored to the patient's specific underlying disease. These theranostic probes allow for molecular characterization of cells and cancer cells *in vivo*, i.e., within the whole body, early detection of disease, disease staging, assessment of tumoral molecular heterogeneity by imaging, therapy selection, treatment planning, and subsequent targeted and tailored treatment based on the diagnostic molecular imaging results. The diagnostic part further allows for early assessment of treatment response and detection of recurrence, long before there are measurable anatomical changes, and plays a critical role in the differentiation between progression and pseudo-progression. The use of dosimetry to predict and determine the specific radiation absorbed doses by the tumor, organs at risk, and healthy tissues of every individual patient ensures the desired goals of efficacy and safety of the treatment. Advances in dosimetric software like voxel-based dosimetry will make dosimetry-guided targeted radionuclide therapies (TRT) more feasible. In pursuing a more personalized medicine, radiotheranostic is slowly moving from a fixed schedule of treatment cycles and dose activity to a more individualized approach based on the patient's specific tumor burden, biology, and dosimetry, which will allow immediate adjustment for toxicity and individualized decision making.

## Radiotheranostic Pairs

A radiopharmaceutical consists of three components: the radionuclide (with diagnostic and/or therapeutic properties), a chelator (which links the radionuclide to the ligand/probe), and the ligand/probe (which targets a cancer-specific molecular marker on the tumor cell with high affinity). Sometimes, a radionuclide by itself can serve as a radiopharmaceutical without the need for a chelator or radiolabeling. The purest concept of a 'theranostic pair' consists of a chemically and structurally identical (or nearly identical) probe labeled with either a diagnostic or therapeutic radionuclide. This ensures targeting of the same molecular marker for diagnostic imaging and molecular targeted treatment. Radiolabeling is a critical step in the synthesis of a radiopharmaceutical since the receptor binding affinities may be negatively affected by a decreasing degree of similarity between the diagnostic and therapeutic molecule. In that sense, the 'perfect' theranostic pair would be two isotopes of the same element. The prime example is radioiodine, where for instance ^123^I (single photon emitter) or ^124^I (positron emitter) can be used for diagnostics and ^131^I (beta emitter) for treatment of thyroid diseases. These isotopes are chemically 'identical', and only differ in their emissions and physical half-lives, which is favorable for their respective purposes.

The diagnostic counterpart can be performed by employing either single photon emission computed tomography/computed tomography (SPECT/CT) or more commonly used positron emission tomography either with computed tomography (PET/CT) or magnetic resonance imaging (PET/MRI) to obtain molecular diagnostic images. The chosen radiopharmaceutical is either a gamma emitter for SPECT or a positron emitter for PET. Both gamma and positron emitting radiopharmaceuticals have high tissue absorption, a low energy transfer and a long radiation range, resulting in low-level radiation exposure for the patient with optimal imaging condition. In contrast to anatomical imaging like CT or MRI, molecular imaging visualizes tumor molecules and characterizes tumor tissue, function, and biology. This allows not only for disease localization, staging and restaging, but also, and a unique feature of theranostic, the ability to effectively select patients for subsequent TRT based on their chances to have a positive response to therapy. Molecular imaging determines whether there is sufficient expression of the molecular target based on tumor uptake compared to normal tissue and background uptake, and therefore whether the patient will benefit from TRT. This principle indicates that treatment with the same compound will be delivering a tumoricidal radiation dose to the cancer cells.

TRT is a systemic cytotoxic treatment which is applied either intravenously or orally. The ionizing radiation aims directly at the cancer-specific target and induces deoxyribonucleic acid (DNA) double-strand breaks and subsequently organized cell death through apoptosis. Therefore, choosing the most appropriate radionuclide is key. The higher the linear energy transfer (LET) to the target cell, the higher the damage to the target cell and treatment efficacy. Also, the longer the emission range, which is the tissue penetration range, the larger the perimeter of the irradiated tissue area/treated area (measured in microns up to 2 mm). Preferably, a radionuclide with a relatively long half-life (days to 1-2 weeks) is chosen to prolong the therapeutic effect. The most commonly used radioemitters in the clinic are beta particles like ^131^I, Lutetium-177 (^177^Lu), Samarium-153 (^153^Sm), and Yttrium-90 (^90^Y). They are characterized by a high energy transfer to the tumor cell and a short radiation emission range, which is favorable to spare surrounding healthy tissue cells. Treatment with alpha particles like Radium-223 (^223^Ra) has been approved by the US Food and Drug Administration (FDA), others like Actinium-225 (^225^Ac) are being actively researched in human clinical trials. Compared to beta emitters, they are distinguished by a very high LET and an even shorter path length in the dimension of microns (<100 μm). Another group of emitters that have been used in the past for theranostics are Auger electron emitters. However, these radionuclides are generally less effective as they provide very low energy electrons that decay by electron capture. The energy is deposited over a very short distance, so they become most effective strictly intracellularly. Examples of Auger electron emitters are ^123^I, Indium-111 (^111^In), Gallium-67 (^67^Ga), and Technetium-99m (^99m^Tc), which are currently used for SPECT/CT at very low diagnostic doses, but some of them like ^123^I and ^111^In have been introduced in clinical trials at high doses for treatment of thyroid diseases and neuroendocrine tumors (NET), respectively 8.

In addition, radionuclides usually have two or more types of emission with different energy peaks. This characteristic makes certain radioisotopes used for therapy to be suitable for non-diagnostic imaging. This non-diagnostic imaging can be of great utility to obtain post-treatment SPECT/CT imaging to confirm molecular targeting of the treatment, rule out pharmacologic interference and stunning. This is usually the case with beta emitters, which tend to have a certain abundance of gamma emission suitable for post-treatment imaging with SPECT/CT. These scans can also be used for dosimetry to determine the absorbed doses of the tumor(s) and healthy tissues.

## Thyroid Diseases - Radioiodine and Beyond

Thyroid cancer is the most prevalent endocrine malignancy, and the incidence of papillary thyroid carcinoma (PTC) has increased over the past few decades due to improved diagnosis 9. The American Cancer Society estimates 44,280 new cases of thyroid cancer in 2021 in the United States 10. Differentiated thyroid cancer (DTC) accounts for the vast majority of thyroid cancers. They arise from follicular epithelial cells and are divided in PTC (85%), follicular thyroid cancer including conventional (5%) and oncocytic (Hürthle cell) carcinomas (5%), poorly differentiated (<3%) and anaplastic thyroid cancer (<3%) 11.

Radioiodine treatment utilizes the underlying thyroid physiology of using iodide to synthesize thyroid hormones. Iodide is taken up from the blood stream by follicular thyroid cells through the sodium-iodide symporter (NIS) localized in the basolateral membrane. The NIS cotransports two sodium ions along with one iodide ion into the cytosol whereas the sodium gradient serves as driving force 12. The efflux of iodide across the apical membrane into the follicular lumen is mediated by pendrin channels 13. Thyroid peroxidase organifies iodide by oxidation and attachment to thyroglobulin. Iodinated thyroglobulin re-enters the follicular cell via endocytosis, undergoes hydrolysis, and the thyroid hormones T3 and T4 are subsequently secreted into the blood stream at the basolateral membrane 14. Radioiodine is taken up and trapped in the thyroid cell in the same manner as any other iodine molecule. The thyroid cell cannot differentiate between the structurally identical radioactive and non-radioactive molecule. Several members of the radioiodine family are used for diagnostic or therapeutic purposes for hyperthyroidism and differentiated thyroid carcinoma (Table 1).

From a diagnostic point of view, SPECT imaging with ^123^I can be used for diagnosis and planning of radioiodine therapy in cases of DTC, hyperthyroidism like Graves' disease and toxic uni- or multinodular adenomas. PET imaging with ^124^I is also a great diagnostic agent, although not as easily accessible as ^123^I. ^124^I is used for staging and restaging of DTC, although its long half-life of 4 days makes it an excellent theranostic pair for dosimetry. Currently, the doses of radioiodine prescribed for radioiodine treatment of DTC are based on disease risk, disease burden and location of metastatic disease. This can lead to under- or overtreatment with subsequent adverse effects. Lesional dosimetry aids in moving towards an individualized treatment approach in order to maximize outcome and minimize toxicity. The feasibility of dosimetry with ^124^I in predicting and estimating absorbed doses of individual tumor lesions is currently evaluated in various institutions in the United States and Germany (ClinicalTrials.gov identifiers NCT03647358, NCT03841617 and NCT01704586).

From a therapeutic point of view, treatment with ^131^I is given in various settings: In benign hyperthyroidism to ablate uni- or multinodular toxic goiter or whole organ ablation in Graves' disease; in DTC, as adjuvant therapy to irradiate local or distant metastases and thereby reducing the risk of recurrence, or for residual or recurrent disease, or to ablate any normal thyroid remnant after total thyroidectomy to ensure an undetectable thyroglobulin level which subsequently can be used as tumor marker in follow-up 15, 16. The American Thyroid Association (ATA) guidelines from 2015 recommended radioiodine treatment for high-risk patients, selected cases with intermediate-risk, but not in patients with low-risk disease 15. However, two randomized clinical trials from Europe validated the treatment of low-risk patients with a low activity of 1.1 GBq ^131^I 17, 18 and showed that the rate of relapse was not higher in patients who received 1.1 GBq vs 3.7 GBq ^131^I 18. Further large, randomized studies are underway to evaluate the usefulness of radioiodine treatment in low-risk DTC patients (ClinicalTrials.gov identifiers NCT01837745, NCT01398085). In a joint statement of the ATA, the European Association of Nuclear Medicine, the Society of Nuclear Medicine and Molecular Imaging, and the European Thyroid Association, they acknowledged the lack of substantial data pro or contra radioiodine treatment in low-risk DTC patients and concluded that the decision to treat should be made on an individual basis depending on disease risk factors, patient-related factors (concern, co-morbidities) and healthcare setting 16.

Theranostic with radioiodine is an integral part of (re-)staging, therapy, and surveillance of DTC after initial thyroidectomy. Although the prognosis of DTC is usually favorable, recurrence is seen in 5-30% of the cases 19. Treatment of recurrent or metastatic disease is problematic, especially when these tumors have become refractory to radioiodine 20. MEK inhibitors such as selumetinib have shown ability to induce redifferentiation of radioiodine refractory cancer cells and hence increased radioiodine uptake in a small cohort 21. The SEL-I-METRY trial, a multicenter phase II study (European Union Clinical Trials Register identifier EudraCT 2015-002269-47), is underway to shed more light on the redifferentiation potential of selumetinib.

Medullary thyroid cancer (MTC) derives from C-cells which arise from the neural crest and is therefore considered NET. Primary and curative treatment is surgery. In case of progressive, symptomatic disease, systemic therapy with cabozantinib and vandetanib is recommended 22, 23. However, these multikinase inhibitors bear serious adverse reactions and have not shown a significant survival benefit. As MTC are NET, peptide receptor radionuclide therapy (PRRT) with ^90^Y-DOTATOC, which targets somatostatin receptors (SSR), has been evaluated. A phase II trial evaluating response, survival, and long-term safety of ^90^Y-DOTATOC in patients with metastatic MTC and increasing tumor marker calcitonin showed that 29% of patients demonstrated a decrease in tumor marker which was associated with a significantly higher survival 24. Other studies exploring ^177^Lu-DOTATATE 25 and ^177^Lu-octreotate 26 concluded that PRRT can be considered as alternative. However, PRRT has not yet become clinical routine. Lodewijk et al showed expression of prostate-specific membrane antigen (PSMA) in 90% of the 104 included MTC patients 27. This might warrant future TRT with ^177^Lu-PSMA which is currently used to treat prostate cancer (PC) in clinical trials. Interestingly, a recently published case report showed uptake of ^18^F-FDG, ^68^Ga-DOTATATE and ^68^Ga-PSMA in a patient with radioiodine-refractory, poorly differentiated thyroid cancer 28. Rottenburger et al reported the first in man administration of the cholecystokinin-2 receptor agonist ^177^Lu-PP-F11N in patients with MTC. ^177^Lu-PP-F11N showed promising biodistribution with accumulation in MTC lesions 29. Clearly there is an unmet need for treatment of metastatic MTC. Further randomized clinical trials are needed to compare different TRT with current state-of-the-art multikinase inhibitors in patients with MTC.

## Neuroendocrine Tumors - PRRT

NETs are a heterogenous group of rare tumors. The majority are gastroenteropancreatic NETs (GEP-NET) (60-70%) and bronchial NETs (30%). Although considered rare, their incidence is rising, which might be due to more awareness and incidental findings, but also related to rapidly developing diagnostic methods 30. The classification and nomenclature of NET is complex and not uniform as studies have mostly been focusing on organ-specific NETs. The World Health Organization proposed a 'common framework' to classify NETs: Depending on the site of origin, neuroendocrine neoplasia are divided into NETs and neuroendocrine carcinomas (NEC). NETs are further characterized by grading (low, intermediate, and high grade) based on mitotic rate and ki-67 index, which is a marker for cell proliferation 31. The distinctive feature of well-differentiated NETs is the overexpression of SSR on their cell surface 32, 33. Somatostatin is in control of hormone secretion such as glucagon, insulin and growth hormone, and cell proliferation 34. Five subtypes of human SSRs have been identified: 1, 2A, 2B, 3, 4 and 5. The majority of NETs show overexpression of subtype 2, especially 90% of GEP-NETs 35-37. Poorly differentiated NETs and NECs have less or no expression of SSR 37. Somatostatin analogs (SSA) were developed to target SSR and were initially administered to relieve symptomatic burden of functioning NETs. As they also showed an antiproliferative effect, their use was expanded to include non-hormone-secreting NETs 38, 39. SSA can be radiolabeled to image SSR expression for theranostic purposes. The diagnostic counterpart for SPECT imaging is ^111^In-octreotide or ^99m^Tc-Hynic-toc. However, SPECT imaging has been largely replaced by PET due to better image quality and its potential for quantification 40. Currently, there are three well established SSA available that are conjugated to DOTA and labeled with ^68^Ga: ^68^Ga-DOTA-1-Nal3-octreotide (DOTANOC), ^68^Ga-DOTA-D-Phe1-Tyr3-octreotide (DOTATOC) and ^68^Ga-DOTA-D-Phe1-Tyr3-Thr8-octreotide (DOTATATE). DOTATATE has a 10-fold higher and selected affinity to SSR2 receptor followed by DOTATOC which also binds to SSR5, whereas DOTANOC is selective towards SSR2, SSR3 and SSR5 41. Despite their different affinity to the various SSR, they all show high affinity to SSR2 and are equally effective in diagnostic accuracy with a pooled sensitivity of 93-96% and specificity of 85-100% 42-50 making PET imaging with ^68^Ga-labeled SSA a powerful tool. In a systematic review and meta-analysis on the impact of ^68^Ga-SSA PET on patient management including 1,561 patients, a management change due to PET/CT findings was seen in 44%. Most interestingly, 77% were intermodality changes, meaning a change in type of treatment (e.g., surgery to chemotherapy). Recently, the FDA approved Copper-64 (^64^Cu)-DOTATATE for PET imaging of SSR positive NETs. ^64^Cu has the advantage of a longer half-life (12.7 hours vs 68 minutes of ^68^Ga), which makes a central production with long haul distribution possible. In a phase III clinical trial evaluating ^64^Cu-DOTATATE PET/CT imaging for NET, a 100% sensitivity and 96.8% specificity was found with no adverse events 51. ^68^Ga-DOTATOC has also been FDA approved based on two clinical trials 52, 53, however, it has no commercial partner yet.

The therapeutic counterpart uses the same SSA radiolabeled with beta emitters like ^177^Lu and ^90^Y or more recently with alpha emitters such as ^225^Ac. Treatment with PRRT has been employed for a quarter of a century now. A magnitude of clinical trials and studies evidence the efficacy of PRRT in terms of decrease in tumor size and tumor marker, symptomatic relief, overall improvement of quality of life, and increase in overall survival (OS). In general, PRRT is known to have a safe profile and possesses little side effects. However, different radioisotopes have different safety profiles. Renal failure due to radiation induced inflammation and fibrosis of the kidneys has been reported, especially when using ^90^Y, but less since the introduction of nephroprotection with amino acids. Hematological toxicity is usually mild and in the majority of cases reversible 54-58. The NETTER-1 clinical trial was the first prospective randomized phase III trial in midgut NETs comparing treatment with high dose SSA vs a combination of normal dose SSA and PRRT with ^177^Lu-DOTATATE. At the data-cutoff date, the median progression free survival (PFS) had not been reached in the PRRT group vs 8.4 months in the SSA group, meaning a 79% lower risk of disease progression or death in the PRRT arm. Partial response of 17% was seen in the ^177^Lu-DOTATATE arm vs 3% in the SSA group. The patients reported a significant improvement in quality of life compared with high-dose SSA. Adverse events were higher in the PRRT group, however only mild in nature including nausea and vomiting due to the concomitant infusion of nephroprotective amino acids, fatigue or abdominal pain, and diarrhea. Hematotoxicity grade 3-4 was low and included neutropenia, thrombocytopenia, and lymphopenia in 1%, 2%, and 9% of patients, respectively, vs none in the SSA arm 59. This game changing study led to the FDA and European Medicines Agency approval of ^177^Lu-DOTATATE for unresectable or metastatic, progressive, well-differentiated, SSR-positive NETs in 2018. As median PFS and OS had not been reached at the data-cutoff date, final analyses are forecasted either after 158 deaths or 5 years after randomization of the last patient and are anticipated soon.

Currently, research is shifting towards PRRT with alpha emitters. Beta emitting radionuclides have a longer range and hence penetration, which impacts adverse events. Alpha emitters not only have a short range but also deliver a high linear dose to the tumor cells, potentially increasing efficacy. Patients with GEP-NET frequently have liver-dominant disease. Liver-directed treatment can deliver a high tumoricidal dose to the hepatic metastases while sparing surrounding healthy liver tissue. A first in human study showed promising results with liver targeted treatment with intraarterial application of Bismuth-213 (^213^Bi)-DOTATOC 60. Other strategies include the use of somatostatin antagonists as antagonists seem to bind to more sites on receptors, even though they are not internalized into the cell. SSR antagonists have a favorable pharmacokinetic and better tumor visualization than agonists 61, 62. The SSA antagonistic theranostic pair ^68^Ga-NODAGA-JR11 and ^177^Lu-DOTA-JR11 (^177^Lu-OPS201) are currently evaluated in phase I/II multicenter studies (ClinicalTrials.gov identifiers NCT02592707, NCT02609737).

## Prostate Cancer - PSMA and GRPR

PSMA is a transmembrane glycoprotein which is highly overexpressed in prostate cancer (PC) 63. Despite the suggestive name, PSMA is also expressed on various other cancers including breast 64 and renal cell cancer 65. High expression is correlated with advanced, high-grade, androgen-independent disease 66. PSMA-targeted compounds are now the most widely used PET radiotracers for staging in intermediate- and high-risk disease and at biochemical recurrence, especially with low prostate specific antigen (PSA) level 67-69. Once conjugated with the chelator DOTA, they can be labeled with positron emitters like ^18^F and ^68^Ga for diagnostics and with beta (^90^Y, ^177^Lu) or alpha emitters (^213^Bi, ^225^Ac) for therapy. ^68^Ga-labeled PSMA inhibitors such as PSMA-11, PSMA-I&T (imaging and treatment) and PSMA-617 are the most commonly used. However, ^18^F-labeled tracers have the advantage of a slightly longer half-life (110 minutes) which allows a wider distribution along with a high yield production in a cyclotron facility. As highly anticipated, the FDA just recently approved the ^18^F-labeled PSMA PET agent DCFPyL 70. PSMA-617 is the most studied ligand for therapy as it showed favorable pharmacokinetics with high internalization, tumor retention and rapid renal clearance 71. Treatment with ^177^Lu-PSMA for metastatic castration resistant PC (mCRPC) has been introduced nearly a decade ago. Results from prospective phase II clinical trials were pivotal with a decline of PSA levels of greater than 50% in 45% of patients whereas any decrease was observed in 60% of patients. Treatment response was registered in up to 82% according to Response Evaluation Criteria in Solid Tumors (RECIST) and Positron Emission Tomography Response Criteria in Solid Tumors (PERCIST) criteria. PFS and OS were improved, and patients reported pain relief and increased quality of life. Treatment side effects were mild, including fatigue, nausea, and pain. Hematological toxicity was also reported mild and reversible in 90%. However, due to the high uptake in salivary glands, xerostomia was common, however, transient 72-76. The TheraP study, a randomized phase II trial, compared treatment with ^177^Lu-PSMA-617 to chemotherapy with cabazitaxel. A higher PSA response rate (66% vs 37%) and lower adverse events (33% vs 53%) were seen in the ^177^Lu-PSMA-617 arm compared to cabazitaxel 77. In contrast, the VISION trial compared treatment with ^177^Lu-PSMA-617 and standard of care (SOC) to SOC alone, i.e., no active chemotherapy regimen in the control group, in PSMA-positive mCRPC patients who had previously received treatment with next-generation androgen receptor signaling inhibition (abiraterone, enzalutamide, etc.) and one or two prior lines of taxane chemotherapy. The first results of this international, randomized, open-label phase III study were just presented at the annual conference of the American Society of Clinical Oncology (ASCO) in the beginning of June 2021: The treatment with ^177^Lu-PSMA-617 plus SOC showed a 60% reduced risk of progression (median PFS ^177^Lu-PSMA-617 and SOC vs SOC: 8.7 vs 3.4 months), and a 38% reduced risk of death (median OS ^177^Lu-PSMA-617 and SOC vs SOC: 15.3 vs 11.3 months). The objective response rate was significantly higher in the ^177^Lu-PSMA-617 plus SOC arm than in the SOC arm (29.8% vs 1.7%), as was the disease control rate (89.0% vs 66.7%) and time to first symptomatic skeletal event (11.5 vs 6.8 months). Despite a higher rate of high-grade (grade 3 - 5) ^177^Lu-PSMA-617 related adverse events, the treatment was well tolerated. Adverse events included bone marrow suppression, xerostomia, nausea and vomiting 78. The sponsoring company announced that they will file for regulatory approval for ^177^Lu-PSMA-617 in the US and Europe.

Alpha emitting PSMA agents for treatment have been a field of active research in the last years. In a first in man study, ^225^Ac-PSMA-617 achieved biochemical and radiological complete remission with low hematotoxicity but severe xerostomia, which remains the dose-limiting toxicity 79. A retrospective analysis of ^225^Ac-PSMA-617 TRT in 73 patients showed a decline in PSA greater than or equal to 50% in 70% of patients, which was associated with a longer OS. The median PFS was 15.2 months and OS was 18 months. Xerostomia was seen in 85% of patients but did not lead to discontinuation of treatment 80. However, the patient population was less pre-treated than in comparable studies 81. More prospective studies comparing alpha emitting agents to chemotherapy are needed.

Gastrin releasing peptide receptor (GRPR) has emerged as a promising novel target. It is highly expressed in PC and shows only low expression in benign prostate tissue 82. Initially, GRPR agonists were synthesized but due to their internalization into the cell, caused side effects. Inspired by research on SSR antagonists, research shifted towards GRPR antagonists. The most studied and promising compound is ^68^Ga-RM2 which has been evaluated for initial diagnosis 83, 84 and biochemical recurrent disease 85, 86 and showed promising results with high tumor uptake and favorable biodistribution. However, one caveat is that GRPR imaging might be limited in patients with hormonal treatment as this may affect GRPR expression in the tumors 87. More prospective trials with larger cohorts using GRPR antagonists are needed, especially in comparison to the currently most widely used ^68^Ga-PSMA-11, to better understand their relationship and potential predictive value of tumor biology and aggressiveness in different types and stages of PC.

Several theranostic pairs were investigated preclinically, however the first compound explored in human is ^177^Lu-RM2 88. Four patients with mCRPC without any further treatment options were treated with ^177^Lu-RM2 after prior confirmation of GRPR expression through ^68^Ga-RM2 PET/CT. The highest absorbed doses were seen in bone lesions, followed by lymph node and soft tissue metastases and were all therapeutically relevant. The highest physiologic uptake was measured in the pancreas, which became the dose limiting organ 88. A phase I/IIa open-label, multicenter trial is currently underway evaluating biodistribution, dosimetry and safety, tolerability, and antineoplastic activity of the GRPR antagonist ^177^Lu-NeoB in patients with GRPR expressing, metastatic solid tumors (ClinicalTrials.gov identifier NCT03872778).

## Bone Seeking Pseudotheranostic Pairs

Many cancers metastasize to the skeleton causing pain, pathologic fractures, and spinal cord compression thus driving morbidity and mortality. Osteoblastic metastases are characterized by increased bone metabolism which can be targeted by bone seeking radiopharmaceuticals such as ^99m^Tc-biphosphonates for SPECT and sodium fluoride-18 (NaF^18^) for PET imaging, and subsequent treatment labeled to an alpha or beta emitter. Bone seeking radiopharmaceuticals accumulate in the bone depending on the degree of osseous metabolism, meaning the higher the bone turnover, the higher the accumulation of the radiopharmaceutical, hence higher affinity to osteoblastic metastases than healthy bone 89. Bone palliation has been first treated with ^32^P and subsequently other beta emitters such as Strontium-89 (^89^Sr), Rhenium-186-hydroxyethylidene diphosphonate (^186^Re-HEDP), ^153^Sm-ethylenediamine tetramethylene phosphonate (^153^Sm-EDTMP), ^177^Lu-EDTMP, and the alpha emitter ^223^Ra. Bone seeking agents can be grouped into calcium analogs like ^89^Sr and ^223^Ra, and radionuclides labeled to a phosphate like ^186^Re-HEDP and ^153^Sm-EDTMP. For radiolabeled phosphates and ^89^Sr, bone palliation has been shown to occur 2 - 10 days after treatment with overall pain relief response rate of 59 - 86%. However, survival is not significantly impacted 90-97.

^223^Ra-dichloride is the currently most widely and commonly used bone targeted treatment. It has been FDA approved in 2013 for symptomatic bone metastases in mCRPC without known visceral metastases. ^223^Ra is a calcium analog, which forms complexes with the bone mineral hydroxyapatite in areas of high bone metabolism. It therefore does not target the tumor cell itself but rather accumulate in between them. The short range of the alpha emitter allows high LET to the tumor stroma, causing DNA double-strand breaks, but minimizes damage to the surrounding normal tissue, especially the bone marrow 98. The landmark study which led to approval was the ALSYMPCA trial, a randomized double blinded multicenter phase III clinical trial. Patients, before or after chemotherapy with docetaxel, were stratified in a treatment group which received six cycles of ^223^Ra with an interval of four weeks, and a placebo group receiving best supportive care. The key results were a significant delay in time to first symptomatic skeletal event in the ^223^Ra group vs placebo group (15.6 vs 9.8 months) and better OS (14.9 vs 11.3 months). Adverse events were seen slightly more frequently in the placebo than in the ^223^Ra group (96% vs 93%). ^223^Ra related side effects included mainly hematotoxicity, nausea, and other gastrointestinal reactions. Quality of life has been significantly improved in the treatment group 99. PSA showed to be unreliable for response assessment. First treatment effects were reduced bone pain (typically after two weeks) or decreased alkaline phosphatase. Furthermore, the ALSYMPCA trial showed that ^223^Ra was safe and effective when used before or after chemotherapy with docetaxel. However, concurrent ^223^Ra therapy and chemotherapy is not recommended. Adding ^223^Ra therapy to abiraterone or enzalutamide has been reported safe, however the combination treatment did not improve bone palliation, on the contrary, was associated with increased incidence of pathologic fractures and mortality 100. A small clinical trial showed that patients who had previously been treated with ^223^Ra and progressed afterwards can be safely re-treated without serious drug-related adverse events in a two year follow up after re-treatment 101.

Although ^223^Ra was only approved for PC, ^223^Ra could target osteoblastic metastases of any malignant origin. A recently published phase II study showed high disease control rate of 49% and tumor response rate of 54% of ^223^Ra combined with hormonal therapy in hormone receptor‐positive, bone‐dominant metastatic breast cancer 102. Osteosarcoma cells are also known to have a high avidity for bone seeking agents, not only at the primary site but also in soft tissue metastases, therefore making ^223^Ra an ideal systemic therapy for patients with metastatic osteosarcoma. A single phase I dose escalation study has been published hitherto showing feasibility and safety of up to six cycles ^223^Ra with minimal hematologic toxicity in a small cohort of 18 patients with high-risk osteosarcoma 103. Further studies are expected.

## Neuroblastoma, Pheochromocytoma, Paraganglioma - mIBG

Neuroblastoma is the most frequent extracranial solid tumor in infancy which derives from primitive neural crest cells from the sympathetic nervous system. These tumors are heterogenous in nature, varying in location, and clinical behavior ranging from spontaneous regression to aggressive, metastatic disease 104, 105. Pheochromocytoma and paraganglioma (PPGL) are also rare tumors arising from the neural crest. Pheochromocytomas originate from the adrenal medulla and paragangliomas from extra-adrenal, the sympathetic or parasympathetic ganglia. As these tumors are very heterogenous, their management is challenging, especially in the advanced, metastatic setting 106-108. The distinct feature of tumors deriving from the neural crest is the overexpression of norepinephrine transporter on their cell membrane. This is found in 90% of neuroblastomas 109 and 50-60% of PPGL, whereas expression might be low in head and neck paragangliomas 110. High expression of norepinephrine transporter is also found in other NETs like GEP-NET, MTC, Merkel cell carcinoma, and ganglioneuroma. Meta-iodobenzylguanidine (mIBG) is a norepinephrine analog which is taken up by norepinephrine transporters and subsequently stored in neuro-secretory granules via vesicular monoamine transporters 1 and 2, thus irradiating the cancer cell when radiolabeled to ^131^I 107. ^131^I-mIBG has been approved by the FDA in late 2018 for the treatment of mIBG positive, unresectable, locally advanced, or metastatic pheochromocytoma or paraganglioma based on the results of an open-label, single-arm, multicenter clinical trial involving 68 patients. Treatment response was achieved in 22% of patients receiving a single dose and increased to 32% in patients who received two doses. Ninety-two percent of patients who received at least one dose, showed partial response or stable disease. The median OS was 36.7 months. Clinical benefit was reflected in a 50% or greater reduction of all antihypertensive medication for at least six months in 25% of patients. Patients without hypertension were not included in this study, however, this might not have an impact on overall oncologic course of disease. The most common side effect was of hematologic nature in 90% of patients, whereas 72% were severe (grade 3-4). Despite the high percentage of severe adverse events, they were transient and resolved without intervention in all but 25% who needed supportive care such as red blood cell and platelet transfusion, granulocyte colony-stimulating factors or erythropoietin for a limited time. In 16% of patients, ^131^I-miBG treatment had to be discontinued due to persistent severe myelosuppression or other non-hematologic adverse reactions such as nausea. Myelodysplastic syndrome was seen in 4% and acute leukemias in 3% 111. Of note is that patients in this study were not prospectively stratified by genetic mutations. Especially succinate dehydrogenase complex iron sulfur subunit B (SDHB) mutations are associated with an unfavorable prognosis, and false negative ^123^I-mIBG scans have been reported 112. Patient preparation for ^131^I-mIBG diagnostic and treatment includes discontinuation of medication that interfere with norepinephrine transporters for at least five half-lives before and seven days after treatment (i.e., blood pressure medication such as combined alpha/beta blocker labetalol and calcium channel blockers, antidepressants, tramadol and pseudoephedrine) to avoid any false negative scans or transporter saturation *a priori*. As mIBG is radiolabeled with ^131^I, thyroid blockade should be given at least 24 hours prior to treatment and ten days afterward.

For neuroblastoma, therapy with ^131^I-mIBG showed promising results with a 30% - 40% response rate, especially in high-risk neuroblastoma with refractory, relapsed or resistant conditions 113, 114. Hematological side effects are commonly seen, often amplified by combined chemotherapy.

As PPGL are also NETs, they express SSR on their cell surface which makes theranostic with ^68^Ga-DOTATATE and ^177^Lu-DOTATATE possible. Especially in patients with metastatic, extra-adrenal primaries or familial PPGL, the sensitivity of mIBG may drop to 53 - 61%. SDHB mutation is the most common germline mutation seen in PPGL. It is characterized by a high malignant transformation (30%), shorter survival (5-year survival rate 36% vs 67% in patients without SDHB mutation) and 20% do not produce norepinephrine 115, 116. These patients might benefit from PRRT. In hereditary forms and germline mutations, the various radiopharmaceuticals for molecular imaging allow for stratification and selection of the right treatment for the patient.

## Future Developments in Theranostics

Active preclinical research has resulted in the development of new molecular targets with high potential in translating into the clinic and application through theranostics. Here, we present just a few of the many novel targets evolved.

### Fibroblast Activation Protein Inhibitors - FAPI-04

A malignant tumor contains not only cancer cells but also non-malignant cells such as inflammatory and vascular cells, and fibroblasts that form the tumor microenvironment, the so-called tumor stroma. Cancer-associated fibroblasts make 90% of the tumor mass and highly express the membrane bound glycoprotein fibroblast activation protein (FAP) in epithelial carcinomas, especially in colorectal, breast, pancreatic and PC 117. FAP is involved in tumor proliferation and escape from immunosurveillance 118, 119. Various small molecule FAP inhibitors (FAPI) have been developed for imaging and treatment with promising results. ^68^Ga-FAPI-02 and ^68^Ga-FAPI-04 showed high uptake in several cancers with the highest uptake in sarcoma, esophageal, breast, lung and cholangiocellular carcinoma 120. Preclinical studies on human pancreatic cancer cells with ^225^Ac-FAPI-04 showed significant tumor growth delay 121. In a first in man study, ^90^Y-FAPI-04 was administered to one patient with metastasized breast cancer and reduced pain medication significantly. Despite the high uptake in the tumors, a fast clearance was observed 122. Improved ligands with a longer tumor retention time are needed to improve tracer pharmacokinetics. Clinical data on treatment with FAPI are yet scarce.

### Neurotensin

Neurotensin is a neuropeptide exhibited by the gastrointestinal and central nervous system as well as the myocardium. Neurotensin receptor 1 has been found to be overexpressed in various cancers such as colorectal, small cell and non-small cell bronchial, breast and pancreatic cancer. A first in man study showed the feasibility of treatment with ^177^Lu-3BP-227 in six patients with pancreatic adenocarcinoma. The therapy was well tolerated with reversible grade 2 anemia as adverse event. One patient achieved partial response and significant improvement of symptoms while the other survived 11 months after treatment with ^177^Lu-3BP-227 123. Currently, a multicenter phase I/II clinical trial is underway to evaluate the safety, tolerability, biodistribution and antitumor activity of ^177^Lu-3BP-227 in solid tumors expressing neurotensin receptor 1 (ClinicalTrials.gov identifier NCT03525392).

### Immuno-agents

The CXC-chemokine receptor type 4 (CXCR-4) is highly overexpressed in various human cancers like leukemia, lymphoma, and multiple myeloma 124. Receptor imaging with ^68^Ga-labeled pentixafor has shown promising results for imaging of multiple myeloma and lymphoma. A first in human study explored ^177^Lu- and ^90^Y-labeled pentixather for the treatment of relapsed multiple myeloma and showed partial and complete response to CXCR4-directed treatment 125.

The anti-CD20 monoclonal antibody rituximab is a standard treatment for B-cell non-Hodgkin lymphoma. In the setting of relapsed or refractory disease, ^131^I-tositumomab and ^90^Y-ibritumomab tiuxetan have demonstrated efficacy 126, 127. However, anti-CD20 resistant disease calls for the necessity of other targets. The CD37 targeting agent ^177^Lu-lilotomab satetraxan has been explored in a phase I/IIa study with an overall response rate of 70% whereas 32% showed complete response 128. Adverse events included transient grade 3 and 4 neutropenia and thrombocytopenia. These very promising results warrant future clinical trials.

Future objectives of theranostics not only include the development of new molecular targets, but also improvement of currently available treatments. The implementation of TRT earlier in the course of disease might improve quality of life as reported side effects from chemotherapies and other targeted systemic treatments are higher (52, 53). Dosimetry estimates cumulated radiation dose and in a personalized medicine fashion will allow for individualized dosing and number of treatment cycles as opposed to current fixed regimen of dose and treatment cycles 129. Currently, TRT mostly aim at stabilizing disease and improving quality of life in the palliative setting. However, a combination of treatments, especially in the early stage of disease might have potential of achieving complete remission. This is currently explored in small-cell lung cancer treated with ^177^Lu-DOTATATE combined with nivolumab (ClinicalTrials.gov identifier NCT03325816) or in mCRPC combining treatment with ^177^Lu-PSMA-617 with pembrolizumab (ClinicalTrials.gov identifier NCT03805594). Other combinations include TRT with chemotherapy and radiation therapy (65).

## Conclusion

In this era of precision medicine in oncology, theranostic is the ultimate example of targeted, personalized diagnostic and treatment as we treat what we see. Each tumor's molecular profile is unique and so should the therapeutic approach be. Theranostic allows for imaging these specific tumor markers and patient selection and stratification so that the most beneficial treatment can be chosen. Active research in the field of theranostic will evolve more novel targets and improve radiopharmacokinetics. In the future, dosimetry will pave the way to an even more personalized treatment approach by moving from empiric standardized doses to individualized treatment doses and cycles. Molecular targeted radioligand therapy will become a major part of daily clinical nuclear medicine.

## Figures and Tables

**Figure 1:**
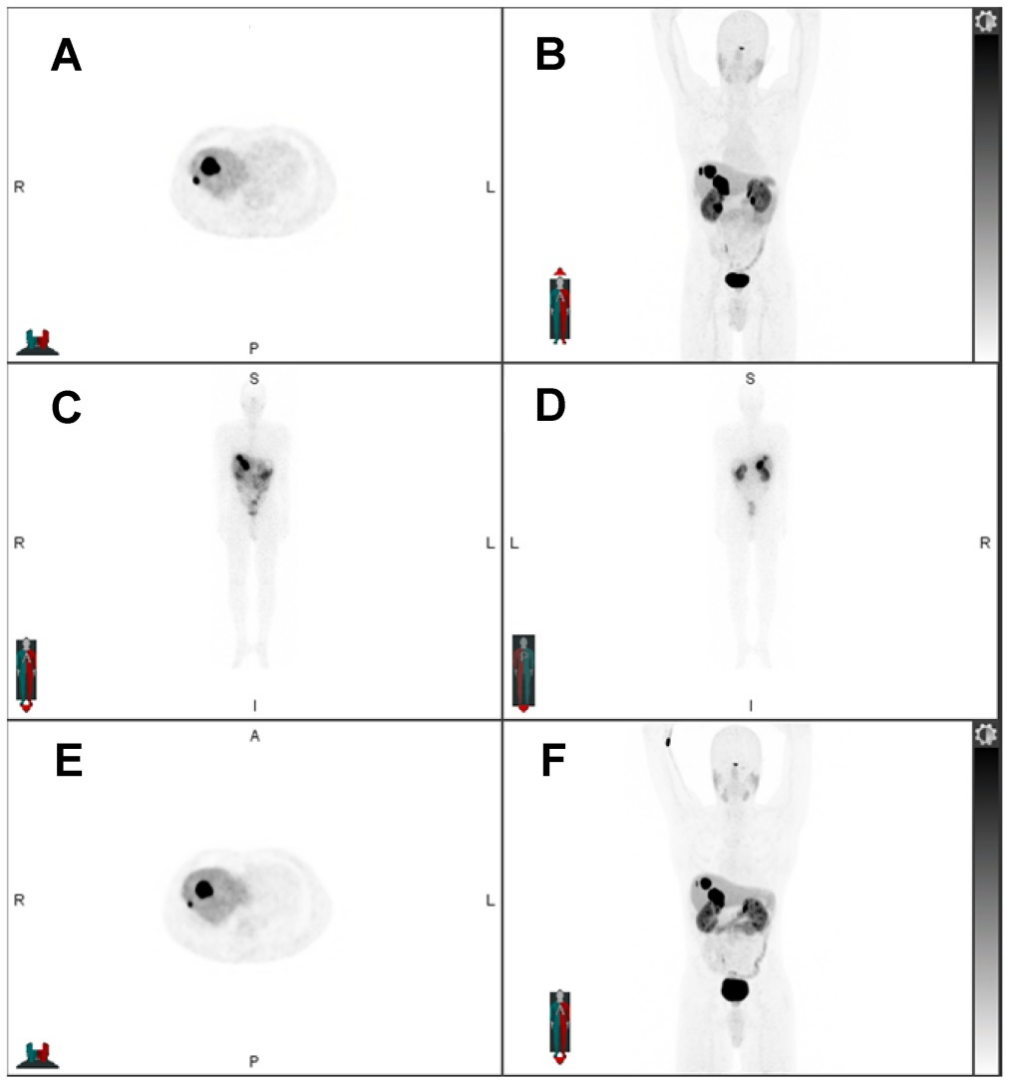
55-year-old man with pancreatic NET, first diagnosed in September 2017 without hormone hypersecretory syndrome. Initial staging was pT3 N1 M1 to the liver with a ki-67 of 18.4%, grade 2. The patient underwent distal pancreatectomy, splenectomy and right hepatic lobectomy, and subsequent chemotherapy with everolimus. Restaging showed progression of liver metastases and chemotherapy with temozolomide and capecitabine was initiated. However, the liver metastases were unresponsive to chemotherapy. The patient was evaluated for PRRT and showed SSR expression. Subsequent treatment with four cycles of ^177^Lu-DOTATATE. Pretreatment ^68^Ga-DOTATATE PET showed liver-only disease: A) axial and B) maximum intensity projection (MIP) ^68^Ga-DOTATATE PET. Post ^177^Lu-DOTATATE treatment SPECT confirmed treatment targets: C) planar anterior and D) posterior view of ^177^Lu-DOTATATE SPECT. Post-therapeutic whole-body SPECT/CT was obtained for treatment evaluation and dosimetric purposes. Interval ^68^Ga-DOTATATE PET after two treatment cycles showed decreased size of liver metastases with central necrosis: E) axial and F) MIP ^68^Ga-DOTATATE PET.

**Figure 2:**
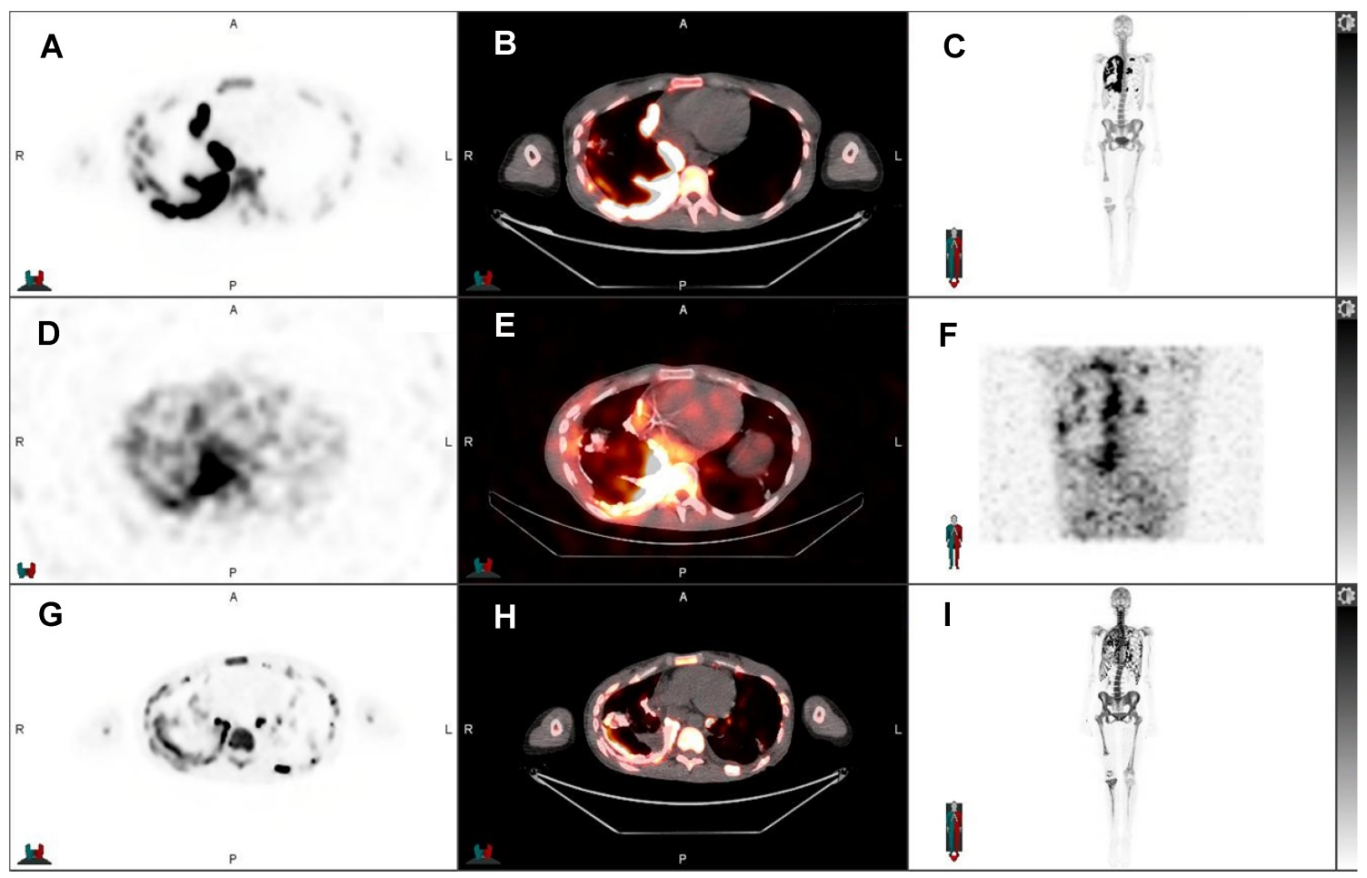
32-year-old woman with progressive metastatic osteosarcoma to the pleura, first diagnosed in February 2019. She underwent resection of the primary tumor in the right knee and multiple lines of chemotherapy. As her disease burden markedly increased with extensive right pleural metastases, few left pleural metastases, and right internal mammary, mediastinal, and hilar nodal metastases, she was referred for ^223^Ra treatment under compassionate care. Pretreatment Na-^18^F PET/CT showed uptake in the pleura: A) Axial Na-^18^F PET, B) axial fused Na-^18^F PET/CT, C) MIP Na-^18^F PET. Post ^223^Ra therapy SPECT/CT evidenced uptake of ^223^Ra in the calcified osteosarcoma lesions in the pleura: D) axial ^223^Ra-SPECT, E) axial fused ^223^Ra-SPECT/CT, F) MIP ^223^Ra-SPECT/CT. The recent Na-^18^F PET/CT after three cycles of ^223^Ra showed stable disease: G) axial Na-^18^F PET, H) axial fused Na-^18^F PET/CT, I) MIP Na-^18^F PET.

**Figure 3:**
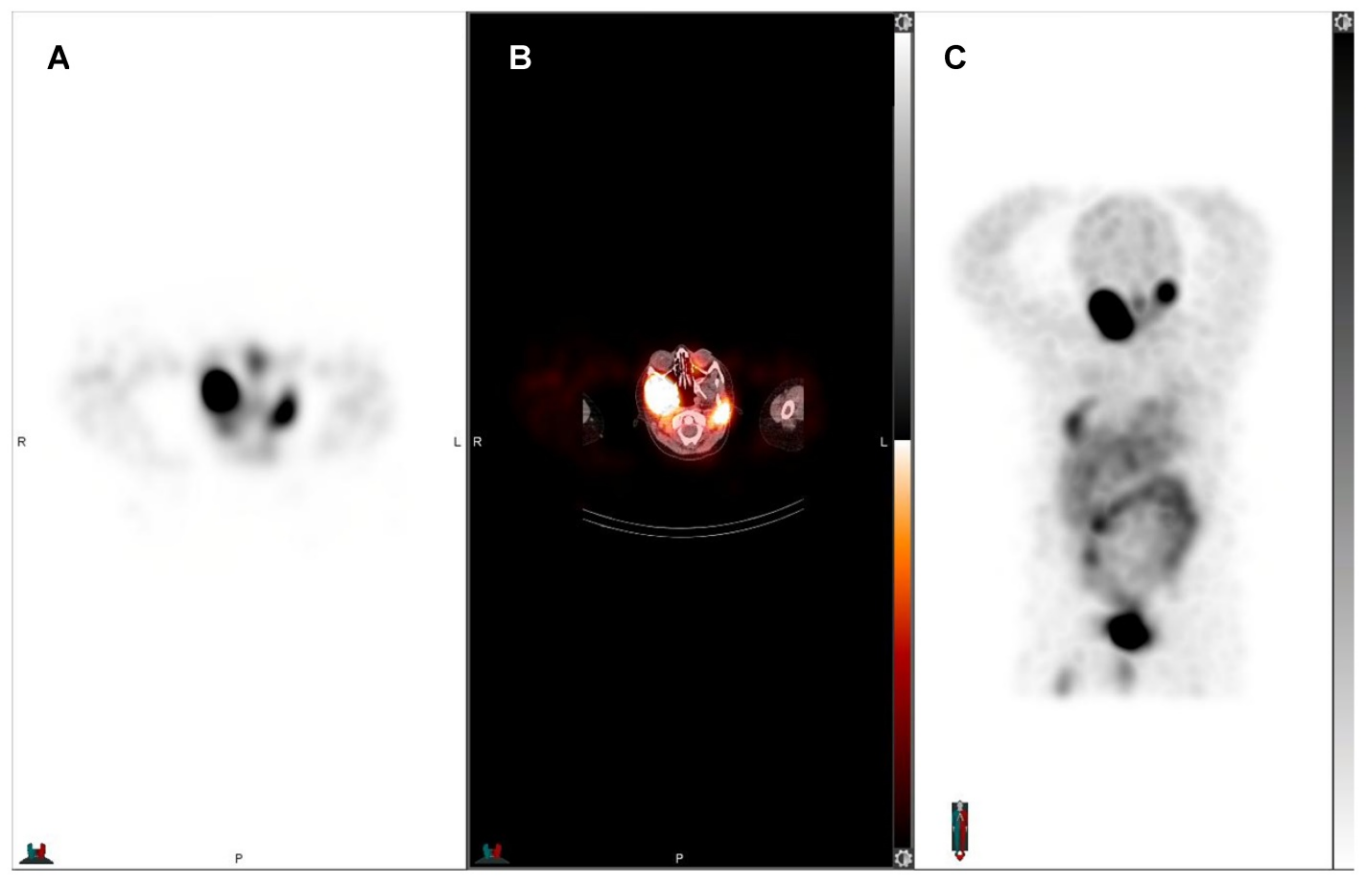
Post-therapeutic ^131^I-mIBG SPECT/CT of an 8-year-old boy with progressive refractory metastatic stage IV ganglioneuroblastoma. After initial ^131^I-mIBG SPECT/CT showed ^131^I-mIBG uptake in the sites of progressive disease in the hemimandible, femur, proximal tibia, and 7^th^ rib, all right sighted, and mastoid left, the patient received ^131^I-mIBG therapy. A) Axial ^131^I-mIBG SPECT, B) axial fused ^131^I-mIBG SPECT/CT, C) MIP ^131^I-mIBG SPECT. The post-therapeutic scan served as treatment verification and was used for dosimetry. ^131^I-mIBG SPECT/CT showed uptake of ^131^I-mIBG in the aforementioned sites of disease.

**Table 1:** Examples of theranostic pairs currently in use.

Disease	Diagnostic	Target	Energy (MeV)	Half-life	Therapy	Target	Energy (MeV)	Max range	Half-life
Differentiated thyroid cancer Hyperthyroidism	^123^I ^124^I	NaI symporter	EC, 0.159 (γ) EC, β^+^	13.22 h 4.2 days	^131^I	NaI symporter	0.606 (β^-^)	2 mm	8 days
Neuroendocrine tumors	^68^Ga-DOTATATE ^68^Ga-DOTATOC ^68^Ga-DOTANOC	SSR (mainly SSR subtype 2)	β^+^	68 min	^177^Lu-DOTATATE ^90^Y-DOTATOC	SSR (mainly SSR subtype 2)	0.498 (β^-^) 2.3 (β^-^)	1.7 mm 11 mm	6.65 days 2.7 days
Metastatic prostate cancer	^68^Ga-PSMA ^18^F-DCFPyL	PSMA	β^+^	68 min	^177^Lu-PSMA ^125^Ac-PSMA	PSMA	0.498 (β^-^) 5.8 (α)	1.7 mm <100 µm	6.65 days 10 days
	^68^Ga-RM2	GRPR	β^+^	110 min	^177^Lu-RM2	GRPR	0.498 (β^-^)	1.7 mm	6.65 days
Bone metastases	^99m^Tc-HDP ^99m^Tc-MDP	New bone formation	141 (γ)	6 h	^223^Ra ^153^Sm-EDTMP	Calcimimetic New bone formation	5-7.5 (α)	<100 µm	11.4 days
Neuroblastoma Pheochromocytoma Paraganglioma	^123^I-mIBG	Norepinephrine transporter	159 keV (γ)	13.3 h	^131^I-mIBG	Norepinephrine transporter	0.606 (β^-^)	2 mm	8 days

EC: electron capture

## Abbreviations

^225^Ac: Actinium-225
ASCO: American Society of Clinical Oncology
DCFPyL: 2-(3-{1-carboxy-5-[(6-^18^F-fluoro-pyridine-3-carbonyl)-amino]-pentyl}-ureido)-pentanedioic acid
DNA: Deoxyribonucleic acid
DTC: Differentiated thyroid carcinoma
DOTANOC: DOTA,1-Nal(3)-octreotide
DOTATATE: DOTA,Tyr(3)-octreotate
DOTATOC: DOTA, D-Phe1, Tyr (3)-octreotide
FAP: Fibroblast Activation Protein
FAPI: Fibroblast Activation Protein Imaging
FDA: Food and Drug Administration
^68^Ga: Gallium-68
GEP-NET: Gastroenteropancreatic neuroendocrine tumor
GRPR: Gastrin releasing peptide receptor
^123/124/131^I: Iodine-123/124/131
^111^In: Indium-111
LET: Linear energy transfer
^177^Lu: Lutetium-177
mCRPC: Metastatic castration resistant prostate cancer
miBG: Meta-iodobenzylguanidine
MTC: Medullary thyroid cancer
NaF^18^: Sodium fluoride-18
NET: Neuroendocrine tumor
NIS: Sodium-iodide symporter
OS: Overall survival
^32^P: ^32^Phosphorus
PC: Prostate cancer
PFS: Progression free survival
PTC: Papillary thyroid cancer
PET/CT: Positron emission tomography/computed tomography
PET/MRI: Positron emission tomography/magnetic resonance imaging
PC: Prostate cancer
PERCIST: Positron Emission Tomography Response Criteria in Solid Tumors
PPGL: Pheochromocytoma and paraganglioma
PSA: Prostate specific antigen
PSMA: Prostate specific membrane antigen
PRRT: Peptide receptor radionuclide therapy
PSMA: Prostate-specific membrane antigen
^223^Ra: Radium-223
^186^Re-HEDP: Rhenium-186 hydroxyethylidene diphosphonate
RECIST: Response Evaluation Criteria in Solid Tumors
^153^Sm-EDTMP: ^153^Samarium ethylenediamine tetramethylene phosphonate
SOC: Standard of care
SPECT/CT: Single photon emission computed tomography/computed tomography
SSA: Somatostatin analogs
SSR: Somatostatin receptor
^99m^Tc: Technetium-99m
TRT: Targeted radionuclide therapy
^90^Y: Yttrium-90
